# Intramedullary versus plate fixation of both bone forearm fractures in skeletally immature patients: a systematic review and meta-analysis

**DOI:** 10.1007/s00590-024-03925-7

**Published:** 2024-04-20

**Authors:** Ahmed Mohamed Ahmed, Elsayed Said, Ahmad Addosooki, Hossam Ahmed Attya, Ahmad Khairy Awad, Emad Hamdy Ahmed, Hamdy Tammam

**Affiliations:** 1 Department of Orthopaedic Surgery and Traumatology, Qena Faculty of Medicine, South Valley University, Kilo 6 Qena-Safaga Highway, Qena, Egypt; 2 Department of Orthopaedic Surgery and Traumatology, Sohag Faculty of Medicine, Sohag University, Sohag, Egypt

**Keywords:** Forearm, Intramedullary, Plating, Children, Adolescents

## Abstract

**Background:**

Both bone forearm fractures (BBFFs) are a common injury amongst the pediatric population. The main indications of surgical fixation are open, irreducible, or unstable fractures. The two most commonly used surgical techniques are closed or open reduction with intramedullary fixation (IMF) and open reduction with plate fixation (PF). The aim of this systematic review and meta-analysis was to determine which fixation method is superior for BBFFs.

**Methods:**

PubMed, Scopus, Web of Science, and CENTRAL were searched to identify studies comparing IMF and PF. We extracted data on union rates, complications, early hardware removal rates, reoperation rates, and radiographic, clinical, and perioperative outcomes.

**Results:**

Sixteen studies were included in the analysis, with a total of 922 patients (539 IMF and 383 PF). Similar union rates were achieved by both fixation technique. IMF was associated with a higher incidence of symptomatic hardware, and early hardware removal. Better restoration of the radial bow was observed with the PF group, especially in older children and adolescents. The rate of excellent function was comparable between groups, whereas better cosmesis was reported with the IMF group. Despite shorter fluoroscopy time and immobilization time, PF demonstrated longer tourniquet time, operating time, and hospital stay compared to IMF.

**Conclusions:**

We found no significant difference between IMF and PF in terms of union rates and functional outcomes taking in consideration the merits and demerits of each technique. High-quality randomized controlled trials are, therefore, necessary to determine the superiority of one fixation technique over the other.

**Level of evidence:**

III.

## Introduction

Both bone forearm fractures (BBFFs) are a common injury amongst the pediatric population accounting for approximately 3–5% of all fractures and 30% of upper extremity fractures [1]. Management of BBFFs is controversial as regards time to operate, acceptable reduction, and age-related remodeling capacity. Most pediatric BBFFs are amenable to nonoperative treatment with closed reduction and cast application. Only 10% of pediatric BBFFS necessitate surgical fixation [2]. The main indications of surgical fixation are open, irreducible or unstable BBFFs. Despite the favourable results of conservative management, surgical fixation of these fractures has become increasingly more common. For instance, Cruz et al. have shown that the percentage of children treated with surgery increased from 59.3% in 2000 to 70.0% in 2012 [3]. The rising trend of operative management may be attributed to technological advancements, socioeconomic changes, liability concerns, and family and surgeon intolerance of residual deformity [4].

The two most commonly used surgical techniques are closed or open reduction with intramedullary fixation (IMF) and open reduction with plate fixation (PF). Several advantages of IMF have been reported, including better cosmesis, minimal soft tissue dissection, decreased operating time, reliable restoration of length and alignment, high union rates, ease of implant removal, and early functional recovery [5–8]. On the other hand, PF also offers several advantages such as immediate fracture stabilization, anatomic reduction, and restoration of normal radial bow location, and magnitude, considered crucial to forearm rotation [9].

Based on the existing literature [10–12], there is no consensus on which fixation modality would provide superior outcomes in terms of safety and efficacy. Therefore, we carried out a systematic review and meta-analysis of studies comparing union rates, complication rates, and radiographic, clinical, and perioperative outcomes of IMF and PF for management of unstable pediatric BBFFs.

## Materials and methods

### Search protocol and information sources

We conducted a systematic review according to the Preferred Reporting Items for Systematic Reviews and Meta-Analyses (PRISMA) checklist [13]. The protocol was registered at PROSPERO (CRD42023494905). PubMed, Scopus, Web of Science and CENTRAL databases were searched from 2000 until 2023 using the following search terms: forearm fracture, both bone, radius and ulna, pediatric, child, adolescent, skeletally immature, intramedullary, plate.

### Eligibility criteria, study selection, and data items

Retrieved results were imported into Endnote X9 software (Thomson Reuters, New York, NY, USA), where a check for duplicates was conducted. The titles and abstracts of the remaining articles were then screened with the following exclusion criteria:

- Articles published in languages other than English.
- Reviews, guidelines, or classifications.
- Letters to the editor or case reports, small case series or conference papers
- In vitro and animal experiment studies
- Irrelevant studies.

Subsequently, full-text articles of potentially relevant studies were obtained and assessed for eligibility. We included studies that met the following inclusion criteria:

- Randomized or non-randomized, prospective or retrospective studies comparing IMF and PF for management of BBFF in skeletally immature individuals.
- The ability to extract data related to the outcomes used for comparison.
- A minimum sample size of 20 patients.

We extracted the following data from studies that met the inclusion criteria: the name of the first author, year of publication, country of origin, study design, study period, number of participants in each group, participants’ age and gender, fracture characteristics, implants used for surgical fixation, and length of follow-up time.

Primary outcomes:

- Union
- Complication, early hardware removal, and reoperation rates.

Secondary outcomes:

- Radiographic outcomes, including radial bow magnitude, and radial bow location.
- Clinical outcomes, including function, and cosmesis.
- Perioperative outcomes, including tourniquet time, fluoroscopy time, operating time, estimated blood loss, length of hospital stay, and immobilization time.

### Data collection process, and risk of bias in individual studies

**Fig. 1:**
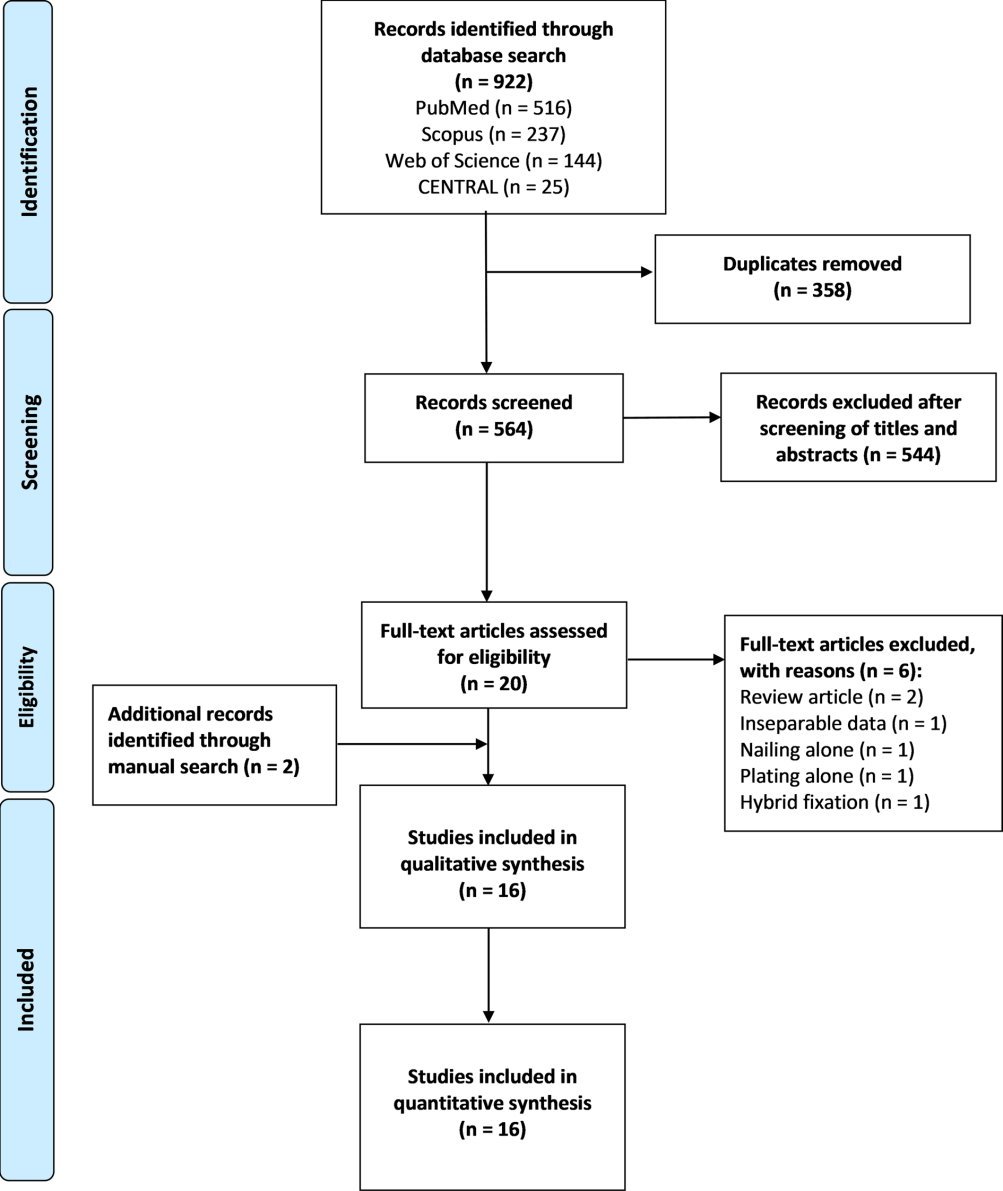
Flow diagram of the study selection process

Two independent reviewers performed the review of the list of potential references and the extraction of data, and a third reviewer was consulted, when necessary, to decide any uncertainties regarding eligibility. The flow diagram for the study selection process is shown in Fig. 1.

The methodological quality of the enrolled studies was evaluated using the Methodological Index for Non-Randomized Studies (MINORS) which consists of eight criteria for non-comparative studies and 12 criteria for comparative studies [14]. Each study scored 0–2 points for each of these items. The methodological quality was determined as follows: a score of 0–12 was considered low quality, 13–18 was considered moderate quality, and 19–24 was considered high quality.

## Summary measures, synthesis of results, and risk of bias across studies

We performed all data analyses using Review Manager version 5.4.1. (Copenhagen: The Nordic Cochrane Centre, The Cochrane Collaboration, 2014). We calculated the odds ratio (OR) with 95% confidence intervals (CIs) for binary outcomes. We calculated the mean difference (MD) with 95% CIs for continuous outcomes. To calculate the overall effect estimate with a 95% CI, we used a fixed-effect model with the method of Mantel–Haenszel when there is no evidence of heterogeneity between studies. Otherwise, a random-effect model with the method of DerSiomonian and Laird was chosen. Heterogeneity between studies was evaluated using the *Q* statistic and *I*^2^ test, which describes the percentage of variability in the effect estimates. Missing values were calculated according to different methods described in the literature [15]. A subgroup analysis was also performed which primarily concerned older children and adolescents. A *P* value of less than 0.05 was used to declare statistical significance. Publication bias was assessed using funnel plots for outcomes reported by at least 10 studies.

## Results

### Study selection

The electronic search yielded 922 references from the four databases. After excluding duplicates and title/abstract screening, we had 20 relevant articles for full-text screening. Fourteen fulfilled the inclusion criteria, and six were excluded; two were review articles, one reported inseparable data, and three looked at either IMF, PF, or hybrid fixation exclusively. The manual search of references imported two additional articles.

### Study characteristics

Sixteen studies [2, 4, 16–29] were included in the analysis, with a total of 922 patients: 539 patients received IMF, and 383 patients received PF. Baseline characteristics of included studies are demonstrated in Table 1. All included studies were retrospective except for one prospective study by Barua et al. [28] Across studies, the mean age ranged from 9.3 to 14.4 years. Eight studies [19, 20, 22–26, 29] were concerned with older children and adolescents aged 10 years or more. The percentage of male patients ranged from 42 to 100%. The follow-up period ranged from 3.4 to 37 months.

**Table 1:** Study characteristics of included studies

First author	Year	Country	Study design	Sample size*	Age*, yr	Male*, %	Follow-up*, mths	MINORS score
Fernandez et al. [16]	2005	Germany	R	45/10	9.3/11.2	71/79	20.6/32.3	17
Smith et al. [2]	2005	USA	R	21/15	9.7/11.3	57/87	NA	15
Carmichael and English [17]	2007	USA	R	15/16	9.7/13.3	47/69	8.5/9.7	19
Ozkaya et al. [18]	2008	Turkey	R	21/14	11.5/13	76/71	37/33.8	17
Reinhardt et al. [19]	2008	USA	R	19/12	12.5/14.4	68/83	13.8/13.8	17
Kose et al. [20]	2008	Turkey	R	21/11	12/13	81/91	22/28	17
Teoh et al. [21]	2009	UK	R	17/17	9.3/9.5	65/65	21.5/31.8	17
Flynn et al. [4]	2010	USA	R	103/44	10.6/12.7	NA	5.1/5.1	18
Shah et al. [22]	2010	USA	R	15/46	13.3/14.1	67/80	NA	15
Zheng et al. [23]	2018	China	R	48/44	13.5/13.4	63/57	14.8/14.9	17
Freese et al. [24]	2018	USA	R	70/32	12.1/14.2	63/69	6/3.4	16
Thapa et al. [25]	2018	Nepal	R	46/30	12.3/12.8	74/80	12/24	17
Topak et al. [26]	2020	Turkey	R	34/18	11.7/13.7	82/94	30.9/29.6	17
Zeybek et al. [27]	2021	Turkey	R	18/19	10.1/11	44/42	5.5/5.8	16
Barua et al. [28]	2021	India	P	20/20	10.4/11	70/70	NA	16
Ishihara et al. [29]	2023	Japan	R	26/26	13.4/13.4	85/100	12.7/12.7	17

*MINORS* methodological items for non-randomized studies, *NA* not available, *mths* months, *R* retrospective, *P* prospective

*Data are presented as intramedullary fixation/plate fixation

In the IMF group, surgical fixation was performed using TENs in 10 studies [2, 4, 16, 17, 22, 24–28], K-wires in six studies [2, 4, 18, 20, 24, 29], rush pins in four studies [2, 17, 18, 24], and Steinman pins in one study [17]. In the PF group, surgical fixation was performed using small DCP in four studies [2, 16, 17, 22], one-third tubular plates in two studies [18, 22], and LCP and LC-DCP in one study [29]. Three studies did not specify the type of IMF implants [19, 21, 23], whereas PF implants were not specified in 10 studies [4, 19–21, 23–28]. In the IMF group, fracture reduction via a mini-open approach was reported by 13 studies in 161 (35.5%) patients [2, 4, 16–22, 24–26, 29].

### Risk of bias within studies

All included studies reported clearly stated aims, endpoints appropriate to these aims, loss to follow-up less than 5%, adequate control groups, contemporary equivalent groups, and adequate statistical analysis. Ten studies [16–21, 23, 25, 26, 29] reported a follow-up period appropriate to the aim of the study, two studies [4, 17] reported consecutive inclusion of patients, and one study [28] reported prospective data collection. None reported unbiased assessment of the study endpoints, or prospective calculation of sample size. As shown in Table 1, the mean MINORS score of the included studies was 16.8 ± 1, ranging from 15 to 19. Accordingly, all included studies had moderate-to-high quality.

### Synthesis of results

#### Union

##### Union time

**Fig. 2:**
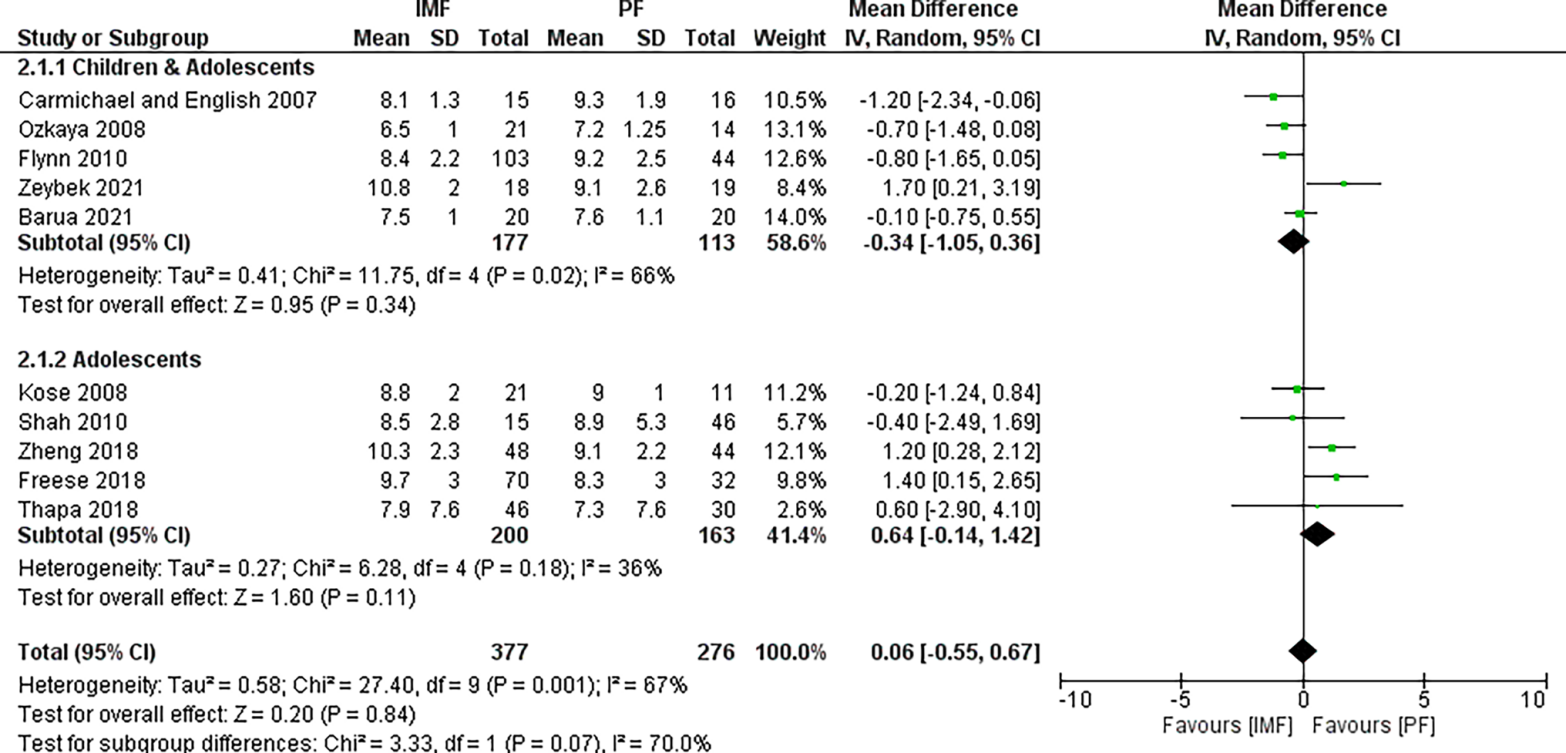
Forest plot of time to union demonstrating no significant difference between IMF and PF

Ten studies [4, 17, 18, 20, 22–25, 27, 28] reported differences in union time with 377 patients in the IMF group, and 276 patients in the PF group (Fig. 2). The majority of studies defined union radiographically by the presence of bridging callus across at least three cortices of bone on standard anteroposterior and lateral radiographs and nontender fracture sites, whereas Ishihara et al. used the modified radiographic union scale in tibial (mRUST) fracture score. The combined MD and 95% CIs was 0.06 (− 0.55 to 0.67). This demonstrates no statistically significant difference between IMF and PF in terms of union time (*Z* = 0.20, *P* = 0.84). Subgroup analysis revealed faster union in older children and adolescents using PF compared to IMF. However, the difference was not statistically significant either.

##### Non-union

**Fig. 3:**
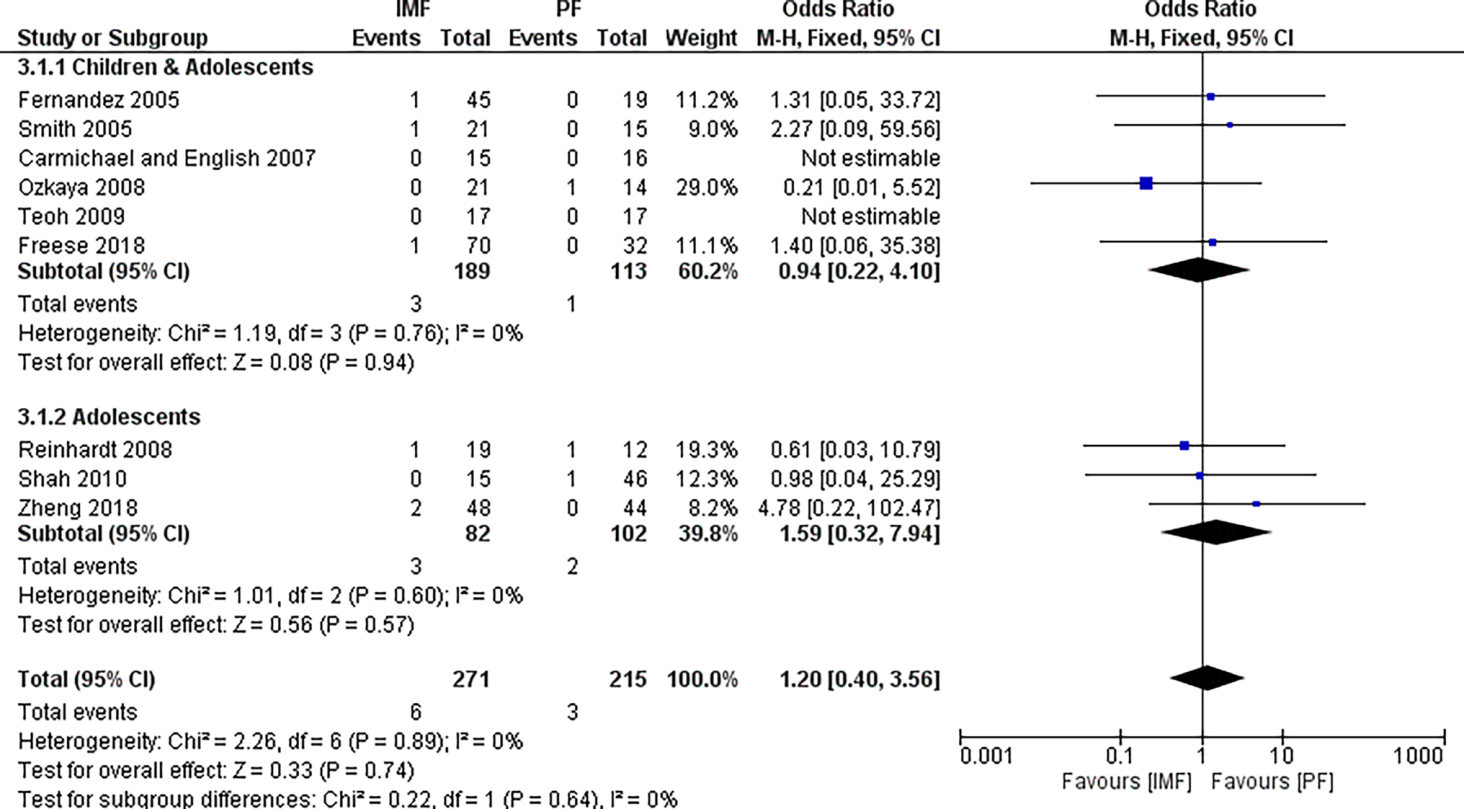
Forest plot of non-union demonstrating no significant difference between IMF and PF

Nine studies [2, 16–19, 21–24] reported differences in the rate of non-union, defined as inadequate consolidation at 6 months, with 271 patients in the IMF group and 215 patient in the PF groups (Fig. 3). The combined OR and 95% CIs was 1.20 (0.40 to 3.56) with no statistically significant difference between IMF and PF in terms of non-union (*Z* = 0.33, *P* = 0.74). By running a subgroup analysis for older children and adolescents, no statistically significant difference was observed between groups either.

##### Delayed union

**Fig. 4:**
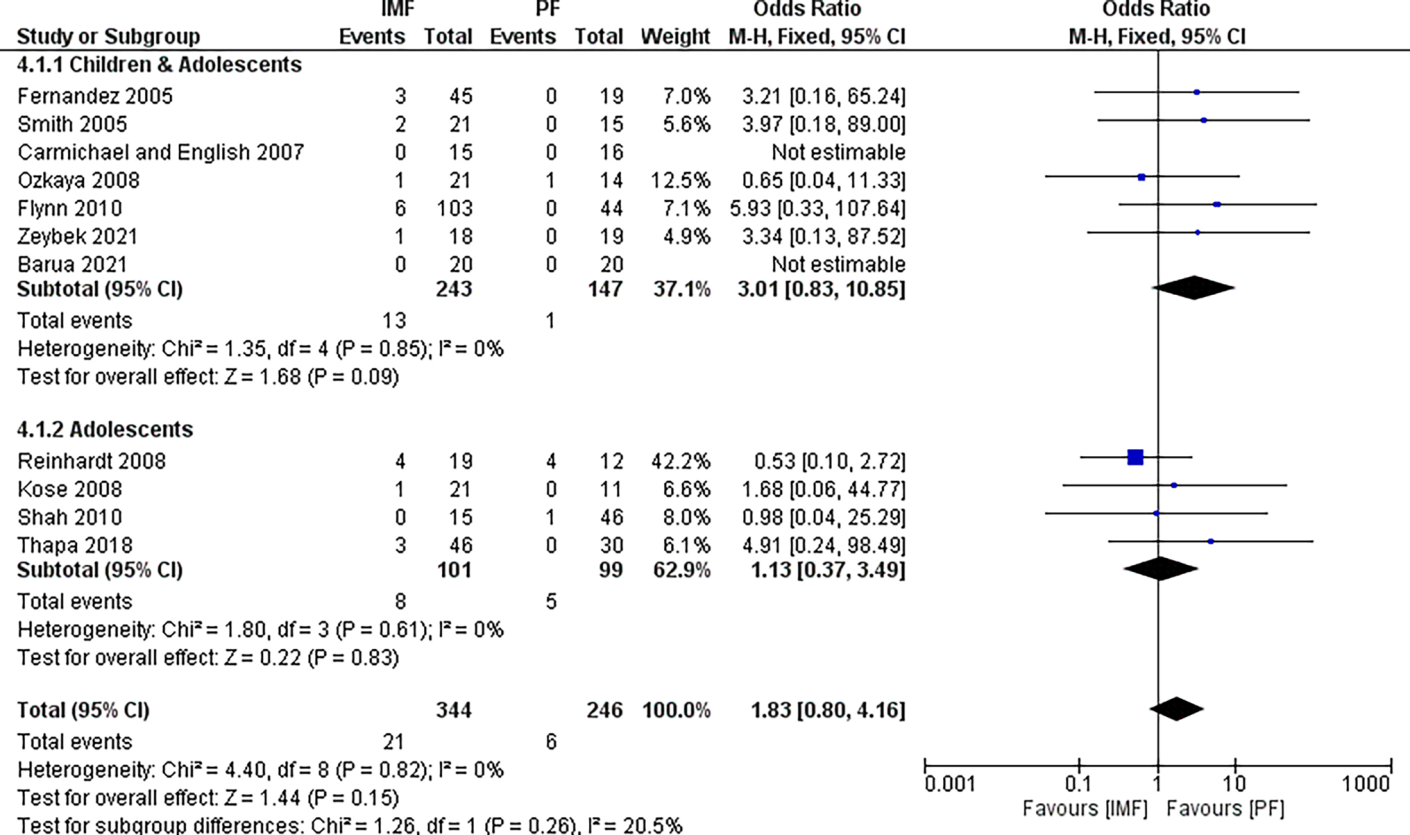
Forest plot of delayed union demonstrating no significant difference between IMF and PF

Eleven studies [2, 4, 16–20, 22, 25, 27, 28] reported differences in the rate of delayed union, defined as inadequate consolidation at 3 to 4 months, with 344 patients in the IMF group and 246 patient in the PF groups (Fig. 4). The combined OR and 95% CIs was 1.83 (0.80 to 4.16) with no statistically significant difference between IMF and PF in terms of delayed union (*Z* = 1.44, *P* = 0.15). By running a subgroup analysis for older children and adolescents, no statistically significant difference was observed between groups either.

#### Complications

In all, eleven complications were selected for analysis, including non-union, delayed union, malunion, refracture, infection, compartment syndrome, neuropathy, limited forearm rotation, limited thumb and finger extension, symptomatic hardware, and hardware failure and migration. The pooled results of these complications are summarized in Table 2. No statistically significant difference could be found between groups in terms of complications except for the higher rate of symptomatic hardware associated with IMF.

**Table 2:** Meta-analysis of complications

Complication	Number of studies	Events/total		Heterogeneity		OR [95% CI]	*P* value
		IMF	PF	*I*^2^	*P* value		
Nonunion	9	6/271	3/215	0	0.89	1.20 [0.40, 3.56]	0.74
Delayed union	11	21/344	6/246	0	0.82	1.83 [0.80, 4.16]	0.15
Malunion	7	2/204	1/175	0	0.70	1.64 [0.20, 13.41]	0.65
Refracture	9	6/290	11/232	0	0.92	0.49 [0.20, 1.18]	0.11
Surgical site infection	15	33/518	18/368	0	0.85	1.32 [0.75, 2.31]	0.34
Compartment syndrome	4	3/169	2/97	0	0.64	0.74 [0.16, 3.47]	0.70
Neuropathy	12	25/337	25/279	0	0.65	0.82 [0.45, 1.47]	0.51
Limited forearm rotation	10	25/240	32/200	0	0.96	0.55 [0.31, 0.99]	0.05
Limited thumb/finger extension	5	4/261	4/135	0	0.64	0.56 [0.17, 1.79]	0.33
Symptomatic implant	11	29/324	2/263	0	0.95	4.32 [1.86, 10.01]	0.01
Implant failure/migration	9	17/249	3/156	0	0.67	2.13 [0.85, 5.35]	0.11

*IMF* intramedullary fixation, *PF* plate fixation, *OR* odds ratio, *CI* confidence interval

#### Early hardware removal

**Fig. 5:**
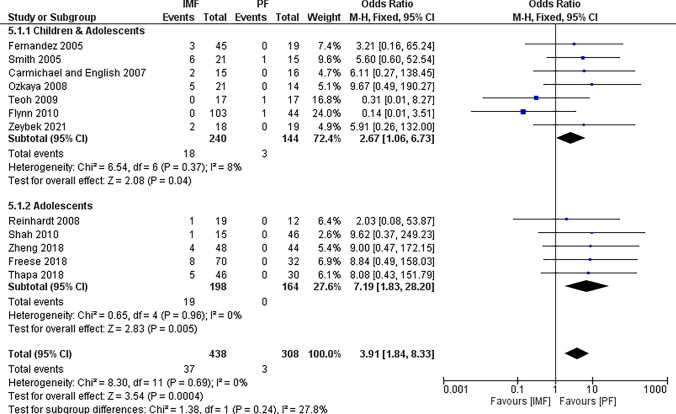
Forest plot of early hardware removal rate demonstrating a significant difference in favor for PF

Twelve studies [2, 4, 16–19, 21–25, 27] reported differences in the early removal rate with 438 patients in the IMF group and 308 patients in the PF group (Fig. 5). The combined OR and 95% CIs was 3.91 (1.84 to 8.33). This demonstrates a significantly higher early removal rates in the IMF group compared to the PF group (*Z* = 3.54, *P* < 0.001). Subgroup analysis revealed similar results in older children and adolescents. Causes of early hardware removal are summarized in Table 3.

**Table 3:** Causes of early implant removal

First author	IMF	PF
Fernandez et al. [16]	3 Infection	None
Smith et al. [2]	4 Irritation, 1 delayed union, 1 bursitis	1 Limited ROM by loose painful plate
Carmichael and English [17]	1 Stitch abscess, 1 backout	None
Ozkaya et al. [18]	4 Migration, 1 bursitis	None
Reinhardt et al. [19]	1 Painful nail tip	None
Teoh et al. [21]	None	1 Irritation by loose ulnar screw
Flynn et al. [4]	None	1 Infection
Shah et al. [22]	1 Bursitis	None
Zheng et al. [23]	4 Irritation	None
Freese et al. [24]	5 Migration, 2 Infection, 1 Irritation	None
Thapa et al. [25]	5 Infection	None
Zeybek et al. [27]	2 Irritation	None

*IMF* intramedullary fixation, *P*F plate fixation, *ROM* range of motion

#### Reoperation

**Fig. 6:**
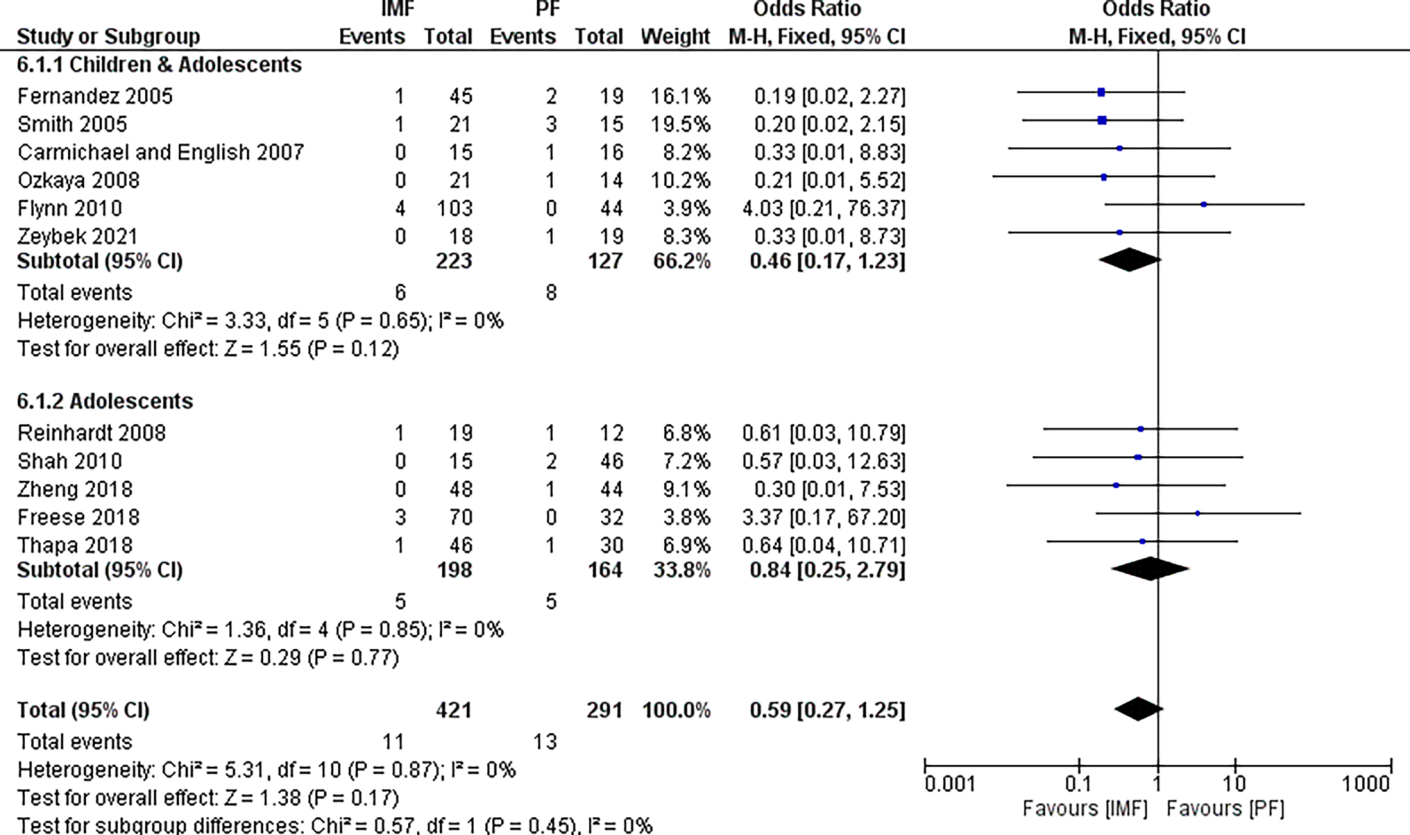
Forest plot of reoperation rate demonstrating no significant difference between IMF and PF

Eleven studies [2, 4, 16–19, 22–25, 27] reported differences in the reoperation rate with 421 patients in the IMF group and 291 patients in the PF group (Fig. 6). The combined OR and 95% CIs was 0.59 (0.27 to 1.35) with no statistically significant difference between IMF and PF in terms of reoperation rate (*Z* = 1.38, *P* = 0.17). By running a subgroup analysis for older children and adolescents, no statistically significant difference was observed between groups either. Causes of reoperation are summarized in Table 4.

**Table 4:** Causes of reoperation

First author	IMF	PF
Fernandez et al. [16]	1 ORIF due to nonunion	1 ORIF due to refracture
Smith et al. [2]	1 ORIF due to nonunion	2 Carpal tunnel release
		1 Fasciotomy due to compartment syndrome
Carmichael and English [17]	None	1 ORIF due to refracture
Ozkaya et al. [18]	None	1 ORIF by locked plate and grafting due to nonunion
Reinhardt et al. [19]	1 ORIF due to refracture	1 ORIF and grafting due to refracture and broken plate
Flynn et al. [4]	4 ORIF due to delayed union	None
Shah et al. [22]	None	1 ORIF due to refracture
		1 Hematoma evacuation
Zheng et al. [23]	None	1 ORIF due to refracture
Freese et al. [24]	2 Corrective osteotomy due to malunion	None
	1 ORIF due to nonunion	
Thapa et al. [25]	1 Adhesiolysis	1 Hematoma evacuation
Zeybek et al. [27]	None	1 ORIF due to refracture

*IMF* intramedullary fixation, *PF* plate fixation, *ORIF* open reduction and internal fixation

#### Radiographic outcomes

**Fig. 7:**
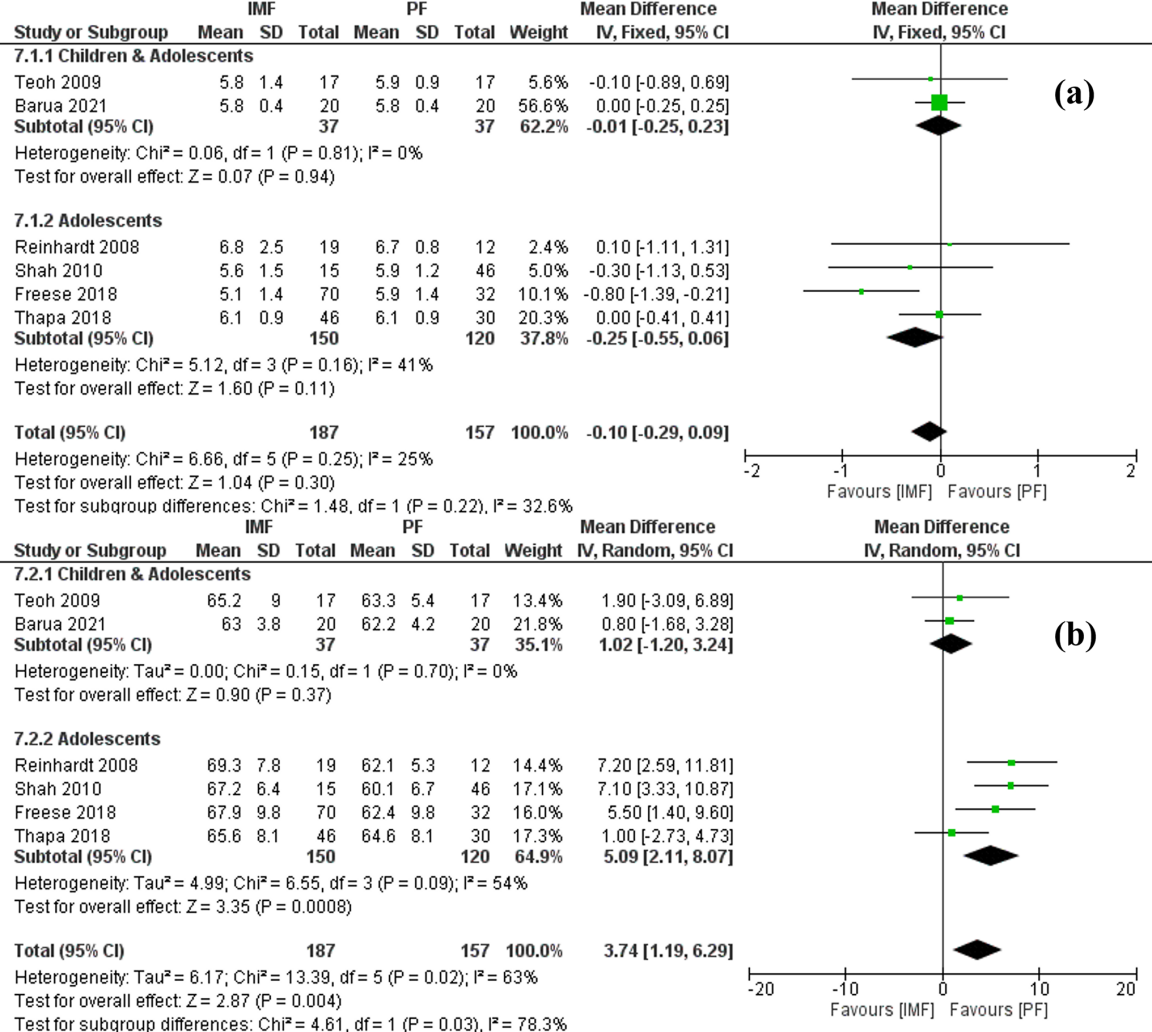
**a** Forest plot of radial bow magnitude demonstrating no significant difference between IMF and PF **b** forest plot of radial bow location demonstrating a significant difference in favor for PF

Six studies [19, 21, 22, 24, 25, 28] reported differences in the magnitude and location of radial bow with 187 patients in the IMF group, and 157 patients in the PF group (Fig. 7). The location and maximum radial bow were measured using the method described by Firl and Wunsch both as a percentage of radial length [30]. The combined MD and 95% CIs of radial bow magnitude was − 0.10 (− 0.29 to 0.09) with no statistically significant difference between IMF and PF (*Z* = 1.04, *P* = 0.30). Subgroup analysis revealed larger bow magnitude in older children and adolescents using PF compared to IMF. However, the difference was not statistically significant either. For radial bow location, the combined MD and 95% CIs was 3.74 (1.19 to 6.29). This demonstrates a significantly more distal location of radial bow in the IMF group compared to the PF group (*Z* = 2.87, *P* = 0.004). Subgroup analysis revealed similar results in older children and adolescents.

#### Clinical outcomes

##### Functional outcome

**Fig. 8:**
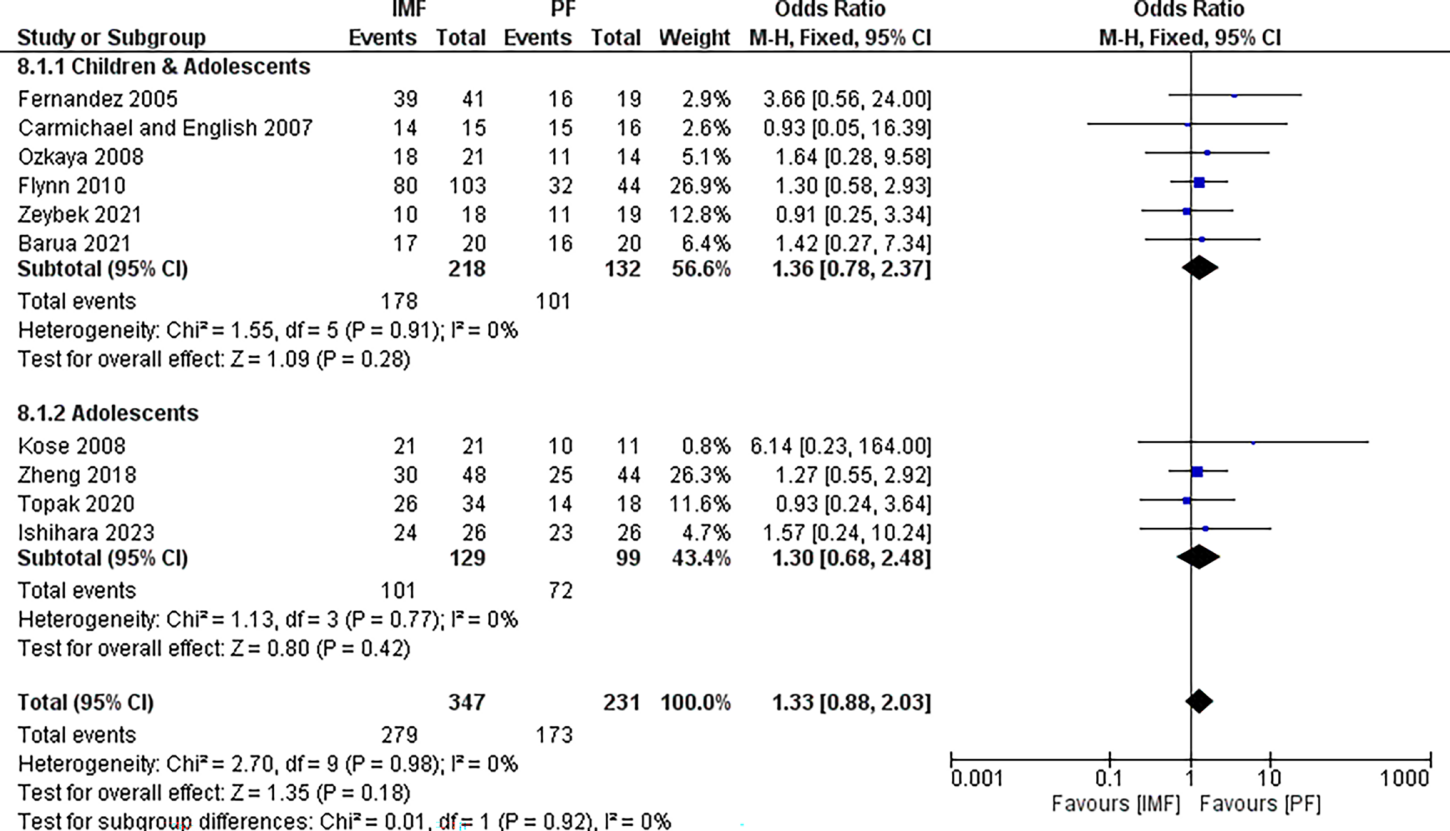
Forest plot of excellent function demonstrating no significant difference between IMF and PF

Twelve studies [4, 16–18, 20, 21, 23, 25–29] reported differences in functional outcome. Nine studies [17, 18, 20, 23, 25–29] used the Price et al. criteria, Flynn et al. [4] used the Children’s Hospital of Philadelphia (CHOP) Forearm Fracture Fixation Outcome Classification, Teoh et al. [21] used the POSNA Outcome Questionnaire, and Fernandez et al. [16] used a subjective evaluation. However, only 10 studies [4, 16–18, 20, 23, 26–29] were suitable for analysis with 347 patients in the IMF group and 231 patient in the PF groups (Fig. 8). The combined OR and 95% CIs of excellent function was 1.33 (0.88 to 2.03). This demonstrates no statistically significant difference between IMF and PF in terms of functional outcome (*Z* = 1.35, *P* = 0.18). By running a subgroup analysis for older children and adolescents, no statistically significant difference was observed between groups either.

##### Cosmesis

**Fig. 9:**
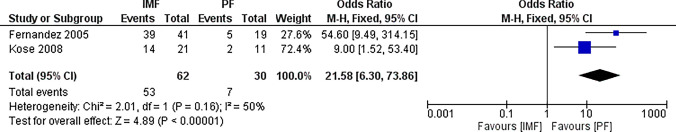
Forest plot of excellent cosmesis demonstrating a significant difference in favor for IMF

Three studies [16, 20, 21] reported differences in cosmetic outcome postoperatively. However, only two studies [16, 20] were suitable for analysis with 62 patients in the IMF group and 30 patient in the PF groups (Fig. 9). The combined OR and 95% CIs of excellent cosmesis was 21.58 (6.30 to 73.86). This demonstrates significantly better cosmetic results in the IMF group (*Z* = 4.89, *P* < 0.001). Teoh et al. [21] also found that patients who underwent IMF had a better Manchester scar score. Table 5 compares the mean scar length between IMF and PF groups.

**Table 5:** Comparing scar length

First author	IMF	PF
Fernandez et al. [16]		
Radial side	2.5 (1.5–3.5) cm	10.2 (7–13) cm
Ulnar side	2.9 (2.0–4.9) cm	10.1 (7–13) cm
Zheng et al. [23]	3.3 ± 0.7 cm	12.9 ± 2.6 cm
Zeybek et al. [27]	3.22 ± 0.88 cm	13.8 ± 2.57 cm

*IMF* intramedullary fixation, *PF* plate fixation

#### Perioperative outcomes

##### Tourniquet, fluoroscopy, and operating time

**Fig. 10:**
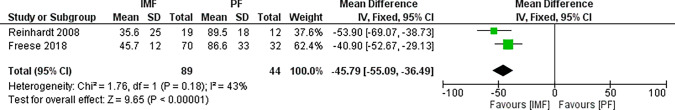
Forest plot of tourniquet time demonstrating a significant difference in favor for IMF

Two studies [19, 24] reported differences in tourniquet time with 89 patients in the IMF group and 44 patient in the PF groups (Fig. 10). The combined MD and 95% CIs was − 45.79 (− 55.09 to − 36.49). This demonstrates a significantly longer tourniquet time in the PF group (*Z* = 9.65, *P* < 0.001).

**Fig. 11:**
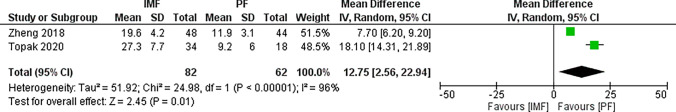
Forest plot of fluoroscopy time demonstrating a significant difference in favor for PF

Three studies [16, 23, 26] reported differences in fluoroscopy time. However, only two studies [23, 26] were suitable for analysis with 82 patients in the IMF group, and 62 patients in the PF group (Fig. 11). The combined MD and 95% CI was 12.75 (2.56 to 22.94). This demonstrates a significantly longer fluoroscopy time in the IMF group (*Z* = 2.45, *P* = 0.01).

**Fig. 12:**
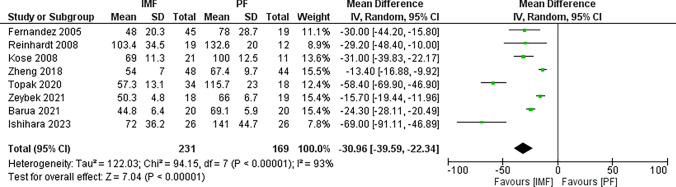
Forest plot of operating time demonstrating a significant difference in favor for IMF

Eight studies [16, 19, 20, 23, 26–29] reported differences in total operating time with 231 patients in the IMF group, and 169 patients in the PF group (Fig. 12). The combined MD and 95% CIs was − 30.96 (− 39.59 to − 22.34). This demonstrates a significantly longer operating time in the PF group (*Z* = 7.04, *P* < 0.001).

##### Estimated blood loss

**Fig. 13:**
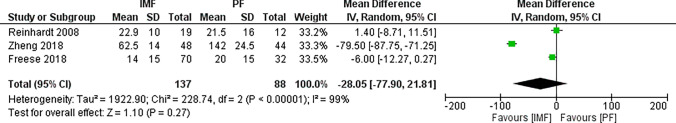
Forest plot of estimated blood loss demonstrating no significant difference between IMF and PF

Three studies [19, 23, 24] reported differences in amount of intraoperative blood loss with 137 patients in the IMF group, and 88 patients in the PF group (Fig. 13). The combined MD and 95% CIs of blood loss was − 28.05 (− 77.90 to 21.81) with no statistically significant difference between groups (*Z* = 1.10, *P* = 0.27).

##### Length of hospital stay

**Fig. 14:**
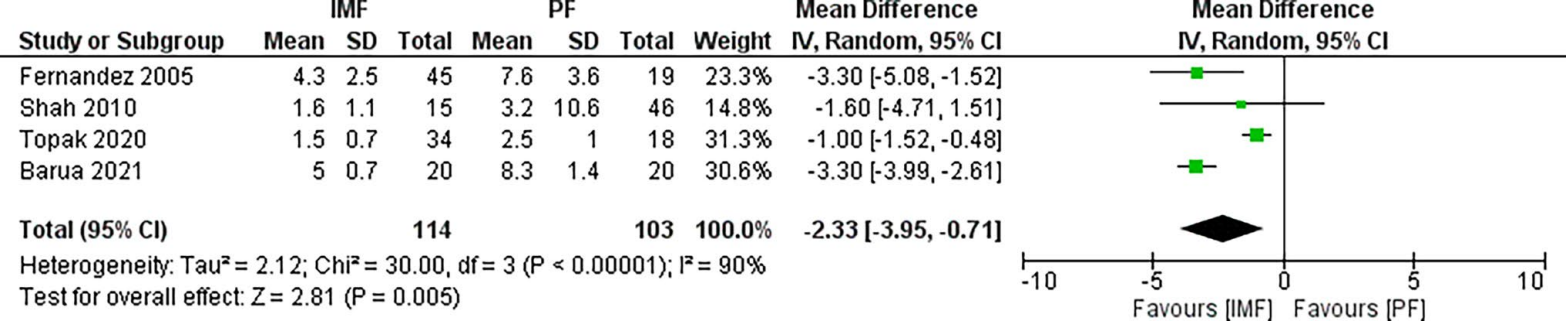
Forest plot of hospital stay demonstrating a significant difference in favor for IMF

Four studies [16, 22, 26, 28] reported differences in length of hospitalization with 114 patients in the IMF group, and 103 patients in the PF group (Fig. 14). The combined MD and 95% CIs was − 2.33 (− 3.95 to − 0.71). This demonstrates a significantly longer hospital stay in the PF group (*Z* = 2.81, *P* = 0.005).

##### Immobilization time

**Fig. 15:**
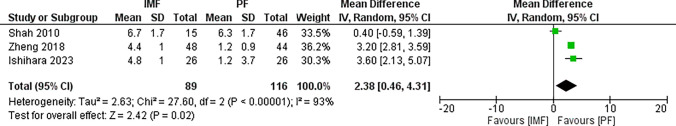
Forest plot of immobilization time demonstrating a significant difference in favor for PF

Seven studies [18, 20–23, 27, 29] reported differences in immobilization time, ranging from 2 to 6.7 weeks in the IMF group, and from 1.2 to 6.3 weeks in the PF group. However, only three studies [22, 23, 29] were suitable for analysis with 89 patients in the IMF group, and 116 patients in the PF group (Fig. 15). The combined MD and 95% CIs was 2.38 (0.46 to 4.31). This demonstrates a significantly longer immobilization time in the IMF group (*Z* = 2.42, *P* = 0.02).

#### Risk of bias across studies

Funnels plots were used to check for publication bias for outcomes reported by at least 10 studies. On visual inspection of the funnel plots, there was a possibility of publication bias found in the published studies measuring symptomatic hardware, early hardware removal rate, and union time. No other variables showed obvious asymmetry.

## Discussion

BBFFs are amongst the most commonly encountered fractures in the pediatric population [31]. Nevertheless, to date, there is no universal agreement on the optimal treatment strategy, especially in unstable fractures where surgical fixation is mandatory [32]. The purpose of this systematic review and meta-analysis was to determine which fixation technique, IMF, or PF, would provide superior outcomes for the management of unstable BBFFs in children and adolescents.

The most important finding of our study was that IMF and PF had similar union rates, and functional outcomes. Better cosmetic results were reported with IMF. Although both techniques had similar complication and reoperation rates, IMF was associated with a higher incidence of symptomatic hardware, and early hardware removal. Despite shorter fluoroscopy time and immobilization time, PF demonstrated longer tourniquet time, operating time, and hospital stay compared to IMF. The advantages of each surgical technique are summarized in Table 6.

**Table 6:** Advantages of intramedullary and plate fixation

IMF	PF
Shorter tourniquet, and operating time	Shorter fluoroscopy time
Shorter hospitalization time	Shorter immobilization time
Faster union in young children	Faster union and better preservation of radial bow in older children and adolescents
Better cosmesis	Less hardware-related symptoms
Easy removal	Less need for early hardware removal

*IMF* intramedullary fixation, *PF* plate fixation

Healing complications in pediatric BBFFs have been shown to be rare by several previous studies [33, 34]. In our study, the mean union time ranged from 6.5 to 10.3 weeks in the IMF group, and from 7.2 to 9.3 weeks in the PF group with no statistically significant difference between fixation techniques [MD (95% CI) = 0.06 (− 0.55, 0.67), *P* = 0.84]. As age is a major determinant of union rates, a subgroup analysis based on patients’ age was performed [35]. Subgroup analysis of patients aged between 10 and 18 years old showed faster union rates with PF. However, the difference did not reach a statistical significance. On the one hand, both IMF and PF demonstrated low non-union rates of 2.2% and 1.4%, respectively. On the other hand, the overall delayed union rate was 6.1% in the IMF group and 2.4% in the PF group. Although not statistically significant, these results suggest that IMF may result in a higher rate of delayed union. Subgroup analysis of older children and adolescents did not reveal additional data as regards non-union and delayed union rates. A mini-open approach was required in 161 of 453 (35.5%) patients undergoing IMF mostly due to soft tissue interposition or inadequate reduction. Although one might postulate that this may interfere with the healing process, our data showed that opening the fracture site did not alter the outcome.

Regarding complications other than non-union and delayed union, the meta-analysis did not favor either technique in terms of malunion, refracture, infection, compartment syndrome, neuropathy, limited thumb and finger extension, hardware failure and migration or reoperation rate. The IMF group demonstrated higher incidence of symptomatic hardware and early hardware removal compared to the PF group. However, unlike plate removal, minimal activity restrictions are necessary after nail removal because of the absence of stress shielding, cortical continuity, and low risk of refracture [36, 37].

It is worth noting that the incidence of limited forearm rotation (more than 10 degrees) was higher in the PF group (16%) compared to the IMF group (10%), and the difference almost reached statistical significance [OR (95% CI) = 0.55 (0.31, 0.99), *P* = 0.05]. It is widely known that anatomical restoration of the radial bow in terms of magnitude and location is critical to normal range of forearm rotation. The average values of radial bow magnitude and location in healthy children are approximately 7.21%, and 60.39%, respectively [30]. In our study, the mean radial bow magnitude ranged from 5.1 to 6.8% in the IMF group, and from 5.8 to 6.7% in the PF group. The mean radial bow location ranged from 63 to 69.3% in the IMF group, and from 60.1 to 64.6% in the PF group. Accordingly, better restoration of radial bow location as well as magnitude was observed with PF group, especially in older children and adolescents. This finding can be explained by the fact that as children approach skeletal maturity, remodelling power declines. However, a statistically significant difference was only observed when comparing radial bow location. An association between residual bow deformity and functional deficit in forearm rotation was suggested by Schemitsch and Richards [9]. However, this was not supported by our findings. Despite better anatomical correction of the radial bow location, PF demonstrated greater percentage of patients with limited forearm rotation, as mentioned above. It should be noted that lack of comparison to the contralateral uninjured arm may have skewed the results.

An important factor in the decision-making process is the functional outcome. The majority of included studies defined excellent outcome according to Price et al. criteria as absence of complaint in strenuous activity and loss of forearm rotation less than 15 degrees [38]. Flynn et al. [4] defined excellent outcome according to the CHOP Forearm Fracture Fixation Outcome Classification as full range of forearm movement and absence of postoperative complications. Fernandez et al. [16] defined excellent outcome as patients being very content. Only one study [21] used a validated scoring system, the POSNA questionnaire, for functional evaluation. The overall rate of excellent function was 80% in the IMF group, and 75% in the PF group with no statistically significant difference. Therefore, we may conclude that the difference in forearm rotation between IMF and PF groups, despite being statistically significant, was clinically insignificant. Furthermore, cosmesis is another important factor to many patients. IMF was clearly the most cosmetic choice in the studies that examined this parameter.

Regarding perioperative outcomes, signifcanlty shorter tourniquet time, and operating time were reported with IMF. However, the estimated blood loss during surgery was similar for the two fixation techniques. Fluoroscopy was used intraoperatively for significantly less time in the PF group due to direct visualization of the fracture site. According to Lu et al., intraoperative parameters may also be affected by the type of orthopaedic surgeon where non-pediatric orthopedists needed significantly longer operating time and fluoroscopy time compared to pediatric orthopedists for intramedullary fixation of pediatric forearm fractures [39]. Although the length of hospital stay was longer in the PF group compared to the IMF group, this parameter can be largely affected by conditions unrelated to the forearm fracture especially in polytraumatized patients. Postoperative cast immobilization time was significantly shorter in the PF group versus the IMF group which may have allowed for earlier functional recovery. A significant heterogeneity was found among studies reporting perioperative data. Therefore, these findings should be viewed with caution as perioperative parameters are surgeon dependent and may vary case by case.

### Limitations

To our best knowledge, this is the most comprehensive systematic review and meta-analysis of the recent literature to determine to what extent the implant type would impact the radiological and functional outcomes of pediatric BBFFs undergoing surgical fixation. Nonetheless, our study had several inherent limitations. First of all, despite our comprehensive search of the literature, we were only able to include observational studies, mostly retrospective in nature, with no mention of blinding or randomization increasing the likelihood of selection bias. Secondly, most included studies had a relatively small sample size with no prospective power calculation to determine the probability of type II error. Thirdly, different studies had different definitions of an excellent outcome, and only one study used a validated functional scoring system. The lack of standardized definitions of outcome measurements may impact the reported overall estimates. Finally, the specific type of intramedullary devices and plates varied across the studies, and it was not possible to assess if specific types may outweigh the others as the number of studies for each type was too small or the type was not specified. Therefore, high-quality standardized randomized controlled trials with larger sample sizes are necessary to determine the superiority of one fixation technique over the other.

## Conclusion

Based on our systematic review and meta-analysis, both IMF and PF are equally effective treatment modalities for BBFFs in skeletally immature patients. While IMF has the advantages of shorter surgical time, and better cosmesis, we may recommend PF for older children and adolescents for earlier union, and better restoration of the radial bow. The merits and demerits of each technique should be discussed thoroughly with a child’s family to reach the best decision possible.
